# Entanglement in photo-ionization process

**DOI:** 10.1038/s41598-024-62198-6

**Published:** 2024-05-18

**Authors:** I. A. Ivanov, Kyung Taec Kim

**Affiliations:** 1 Institute for Basic Science, Center for Relativistic Laser Science, Gwangju, 61005 Korea; 2 Department of Physics and Photon Science, GIST, Gwangju, 61005 Korea

**Keywords:** Atomic and molecular physics, Quantum physics

## Abstract

We report a study of the entanglement between the quantized photon field and an atom arising in the photo-ionization process. Our approach is based on an ab initio solution of the time-dependent Schrödinger equation (TDSE) describing the quantum evolution of a bipartite system consisting of the atom and the quantized electromagnetic field. Using the solution of the TDSE, we calculate the reduced photon density matrix, which we subsequently use to compute entanglement entropy. We explain some properties of the entanglement entropy and propose an approximate formula for the entanglement entropy based on the analysis of the density matrix and its eigenvalues. We present the results of a comparative study of the entanglement in the photo-ionization process for various ionization regimes, including the tunneling and the multiphoton ionization regimes.

## Introduction

Entanglement is an intrinsic feature of quantum mechanics (QM) which is responsible for non-local correlations arising in quantum systems. For a bipartite system *AB*, consisting of two subsystems *A* and *B*, entanglement can be defined as follows^1^. If ${{\mathscr {H}}}_{A}$ and ${{\mathscr {H}}}_{B}$ are the Hilbert spaces for the subsystems *A* and *B* respectively, than the Hilbert space of the combined system *AB* is the tensor product ${{\mathscr {H}}}_{\textrm{A}}\otimes {{\mathscr {H}}}_{\textrm{B}}$. A state of a bipartite system *AB* described by a vector $|\Psi _{AB}\rangle$ is entangled if it cannot be represented as a tensor product of two vectors $|\Psi _{A}\rangle$ and $|\Psi _{B}\rangle$ belonging to ${{\mathscr {H}}}_{A}$ and ${{\mathscr {H}}}_{B}$, respectively. One needs more than one pair of $|\Psi _{A}\rangle$ and $|\Psi _{B}\rangle$ to describe an entangled state, so that:

1 $$\begin{aligned} |\Psi _{AB}\rangle = \sum \limits _i c_i|\Psi _{A_i}\rangle \otimes |\Psi _{B_i}\rangle \, \end{aligned}$$

for some set of vectors $|\Psi _{A_i}\rangle$ and $|\Psi _{B_i}\rangle$. Decomposition (1) is, in general, not unique, essential is that for an entangled state there is more than one pair of $|\Psi _{A_i}\rangle$ and $|\Psi _{B_i}\rangle$ in the sum in the Eq. (1).

The standard prescriptions of QM imply that a system described by a state vector (1) possesses highly nonclassical properties, such as correlations and instantaneous action at a distance, existing even if the parts *A* and *B* of the bipartite system *AB* are sufficiently far away from each other to exclude any causal relations between them. This contradicts the idea of the local realism, i.e., the notion that a system can only be influenced by its nearby surroundings and prompted Einstein, Podolsky and Rosen to put forward, in their famous EPR paper^2^, a conjecture that QM provides only an incomplete description of the physical reality. This paper led to many fascinating discussions of the foundations of the QM, which culminated in the famous Bell inequality^3^, that any theory preserving local realism must obey. The QM violates the Bell inequality and so apparently does the Nature, as it was convincingly demonstrated experimentally^4,5^.

These highly unusual nonclassical properties of the entanglement proved crucial for numerous potential applications, such as quantum teleportation^6,7^, secure quantum cryptography^8^ or the field of quantum computing^1,9^. Various aspects of entanglement arising in different physical situations and systems, such as the electron-electron entanglement in multi-electron atoms in laser fields^10,11^, the entanglement of orbital angular momentum in non-sequential double ionization^12^, or the electron-ion entanglement in ionization process^13–15^ have been studied in the literature. In^16^ exact analytic solutions of the energy eigenvalue equation for the bipartite system consisting of a free electron and a single mode quantized electromagnetic field were proposed. These eigenstates were used to study entangled states of this bipartite system. It was found^16^ that these states are closely related to the number-phase minimum uncertainty states, i.e., the states minimizing product of uncertainties of the photon number and the phase operators^17^. In^18^ dynamic evolution of the bipartite system consisting of a free electron and a single mode quantized photon field interacting on a finite time interval was studied. The Von Neumann entropies^19^ characterizing degree of the entanglement between the electron and the photon subsystems were analyzed and it was found that there is always an entropy production at the end of the process, when electron-field interaction is switched off completely. In the work^20^ the light-matter entanglement in the process of the above-threshold ionization has been studied using an appropriately modified version of the well-known Strong Field Approximation (SFA)^21–26^.

In the present work we report a study of the entanglement between the quantized photon field and an atom arising in the photo-ionization process for various regimes of ionization, including the tunneling and the multiphoton regimes of ionization. Our approach is based on an ab initio solution of the time dependent Schrödinger equation describing quantum evolution of a bipartite system consisting of the atom and the quantized electromagnetic field.

The paper is organized as follows. In Sections Theory and Methods we describe theoretical and numerical techniques that we use. Our results and conclusions are presented in the Sections Discussion and Conclusions. Atomic units with $\hslash =1$, $e=1$, $m=1$ and $c=137.036$ with *e*, *m* being the charge and the mass of the electron and *c* the speed of light, are used throughout the paper.

## Theory

To describe a one-electron atom interacting with the quantized electromagnetic field we use the numerical procedure proposed in the work^27^. For readers convenience we recapitulate the main details of the procedure below.

In the Heisenberg representation the quantized vector potential can be written as^28,29^:

2 $$\begin{aligned} \hat{{\varvec{A}}}({\varvec{r}},t)=\sum \limits _{{\varvec{k}},\lambda } \sqrt{2\pi c^2\over \omega V} g(t) \left( {\varvec{e}}_{{\varvec{k}},\lambda } \hat{a}_{{\varvec{k}},\lambda } e^{-i\omega t+i{\varvec{k}}\cdot {\varvec{r}}} + h.c \right) . \end{aligned}$$

Electromagnetic field is quantized in a finite volume *V*, $a_{{\varvec{k}},\lambda }$ are the photon annihilation operators. The Hilbert space of the bipartite atom and field system is the tensor product ${{\mathscr {H}}}_{\textrm{el}}\otimes {{\mathscr {H}}}_{\textrm{field}}$, where ${{\mathscr {H}}}_{\textrm{el}}$ and ${{\mathscr {H}}}_{\textrm{field}}$ are electron and photon sectors of the Hilbert space, respectively. The factor *g*(*t*) in Eq. (2) is an envelope function which rumps on the atom-field interaction, we will provide more detail about its particular form later.

The computational procedure we employ is based on the well-known fact that the photon Hilbert space is spanned by the Fock states $|N\rangle$- the eigenstates of the operator ${\hat{N}}_{{\varvec{k}},\lambda }={\hat{a}}^{\dag }_{{\varvec{k}},\lambda }{\hat{a}}_{{\varvec{k}},\lambda }$ of the number of photons in the mode ${\varvec{k}},\lambda$. We use only a single mode $({\varvec{k}},\lambda )$ of the quantized electromagnetic field, corresponding to a linear polarization in the *z*-direction and a particular photon frequency $\omega$. We retain thus only one term in the expansion (2) and we will omit, therefore, subscripts ${\varvec{k}},\lambda$ in all the formulas below. We assume, moreover, the dipole approximation in the following. We neglect, therefore, the spatial exponential factors $e^{i{\varvec{k}}\cdot {\varvec{r}}}$ in the calculations. In the Fock states basis the matrix elements of the photon operators in Eq. (2), which we will need in the following, are given by the well-known relations^30^:

3 $$\begin{aligned} \langle N-1|a|N\rangle= & {} \sqrt{N} \nonumber \\ \langle N+1| a^{\dag }|N\rangle= & {} \sqrt{N+1} \, \end{aligned}$$

The initial state of the combined system electron+field at the initial moment of time, $t_0=0$ a.u., is a disentangled product state $\phi _0 \otimes |N_0\rangle$, where $\phi _0$ is the ground atomic state and $|N_0\rangle$ is the initial state of the field.

The quantum evolution of the system is governed by the time-dependent Schrödinger equation (TDSE), where we use the minimal coupling interaction Hamiltonian^28^ to describe the atom-field interaction:

4 $$\begin{aligned} i{\partial |\Phi (t)\rangle \over \partial t}= \left( {\hat{H}}_{\textrm{el}} + {{\hat{{\varvec{A}}}}\hat{{\varvec{p}}}\over c} + {{\hat{{\varvec{A}}}}^2\over 2c^2} \right) |\Phi (t)\rangle . \end{aligned}$$

The setup we are using is similar to the one employed in^18^, where quantum evolution of the bipartite system consisting of a free electron and a single mode quantized photon field was studied. The main difference is that for that system an analytical solution of the TDSE can be obtained^18^, while in the present case of an initially bound atomic electron we have to rely on a numerical procedure which is described below.

We use mixed representation of the quantum operators in Eq. (4). The electron subsystem is described using the more familiar Schrödinger picture, while quantized vector potential operators is described using the Heisenberg form (2). This representation can be obtained from the Schrödinger picture, in which neither electron nor field operators depend on time, by applying the unitary transformation $\exp {\left\{ -i{\hat{H}}_{\textrm{field}}t\right\} }$ generated by the field Hamiltonian ${\hat{H}}_{\textrm{field}}$. This form of the TDSE using the mixed representation is convenient, since it looks similar to the form of the TDSE describing atom-field interaction in the calculations treating electromagnetic field classically. Using this fact, we were able to devise a procedure allowing to solve the TDSE (4), by modifying the numerical codes we have been using to solve the TDSE describing electron evolution in presence of the classical electromagnetic field^31–33^.

In the Eq. (4) $\displaystyle {\hat{H}}_{\textrm{el}}$ is electron Hamiltonian, for which we use the non-relativistic form, $\displaystyle {\hat{H}}_{\textrm{el}}= {\hat{{\varvec{p}}}^2\over 2}+V(r)$. We will consider below two different targets: the hydrogen atom with $V(r)=-1/r$ and the Yukawa atom with with the short-range potential $V(r)=-1.903 e^{-r}/r$. The ground states of both systems are $s-$ states with an ionization potential $|\varepsilon _0|=0.5$ a.u. The non-relativistic description of the electron subsystem, in particular the use of the dipole approximation, is legitimate for the moderate field intensities of the order of several units of $10^{14}{-}10^{15}$ W/cm^2^ that we consider below. To relate the intensity and the photon number $N_0$ for the single-mode Fock state $|N_0\rangle$ which we use as the initial state, we note that in this state the expectation value of the energy flux for the quantum field described by Eq. (2) is $\displaystyle \omega c N_0/V$^30^. The cycle-average of the Poynting vector, computed for the classical monochromatic linearly polarized wave wave ${\varvec{E}}_0 \cos {\omega t}$, is, on the other hand: $\displaystyle cE_0^2/(8\pi )$. The Fock state $|N_0\rangle$ carries, therefore, the same energy flux as the monochromatic wave with the field strength:

5 $$\begin{aligned} E_0=\sqrt{8\pi \omega N_0/V} . \end{aligned}$$

We will use below the value of $E_0$ thus defined as a more familiar and convenient measure of the field strength.

In the process of the quantum evolution driven by the TDSE (4), the atom-field system becomes entangled. Such an entangled wave-function can be written using the completeness of the Fock states in ${{\mathscr {H}}}_{\textrm{field}}$ as:

6 $$\begin{aligned} |\Phi (t)\rangle = \sum \limits _{N=N_0-n1}^{N_0+n2} |f_N(t)\rangle \otimes |N\rangle \, \end{aligned}$$

where $|f_N(t)\rangle$ are vectors from the electron Hilbert space ${{\mathscr {H}}}_{\textrm{el}}$, and the parameters $n_1$, $n_2$ are to be chosen so as to ensure convergence of the expansion (6). More details of the procedure we use to solve the TDSE (4) using the expansion (6) are given in the Section Methods.

## Discussion

### Entanglement entropy

Different measures of entanglement have been proposed in the literature^34–36^. We will use the von Neumann entanglement entropy^19,35^, which for a bipartite system *AB* consisting of two entangled subsystems *A* and *B* can be defined as:

7 $$\begin{aligned} S= - \textrm{Tr} \left[ \hat{\rho }_A\log {\hat{\rho }_A} \right] = - \textrm{Tr} \left[ \hat{\rho }_B\log {\hat{\rho }_B} \right] \, \end{aligned}$$

where $\hat{\rho }_A = \textrm{Tr}_B\hat{\rho }_{AB}$ and $\hat{\rho }_B = \textrm{Tr}_A\hat{\rho }_{AB}$ are reduced density operators describing subsystems *A* and *B*, $\hat{\rho }_{AB}$ is the density operator describing the composite system. Operations $\textrm{Tr}_A$ and $\textrm{Tr}_B$ are partial traces consisting in tracing over all the variables describing subsystems *A* and *B* respectively. Since it is immaterial which subsystem is used to calculate the von Neumann entanglement entropy, we can use the subsystem for which calculations can be performed easier. In our case it is the photon subsystem. The whole system electron+ field is in a pure state described by the state vector $|\Phi (t)\rangle$ in Eq. (6) with the corresponding density operator $|\Phi (t)\rangle \langle \Phi (t)|$. The partial trace with respect to electron variables can be easily computed, giving the following expression for the reduced density matrix describing the state of the field:

8 $$\begin{aligned} \hat{\rho }^F(t)= \textrm{Tr}_{\textrm{el}} |\Phi (t)\rangle \langle \Phi (t)|= \sum _{N_1,N_2} \langle f_{N2}(t)|f_{N1}(t)\rangle |N_1\rangle \langle N_2| \, \end{aligned}$$

where $\rho ^F_{N1,N2}(t)= \langle f_{N2}(t)|f_{N1}(t)\rangle$ is the scalar product of the vectors $|f_N(t)\rangle$ from the electron Hilbert space ${{\mathscr {H}}}_{\textrm{el}}$ occurring in the expansion (6). Matrix elements of $\hat{\rho }^F(t)$ in the basis of the Fock states can, therefore, be easily computed once the TDSE Eq. (4) is solved. The entanglement entropy can then be found as:

9 $$\begin{aligned} S(t)= - \sum \limits _{i} \lambda _i(t)\log {\lambda _i(t)} \, \end{aligned}$$

where $\lambda _i(t)$ are the eigenvalues of the positive definite Hermitian matrix with the matrix elements $\rho ^F_{N1,N2}(t)$.

**Figure 1:**
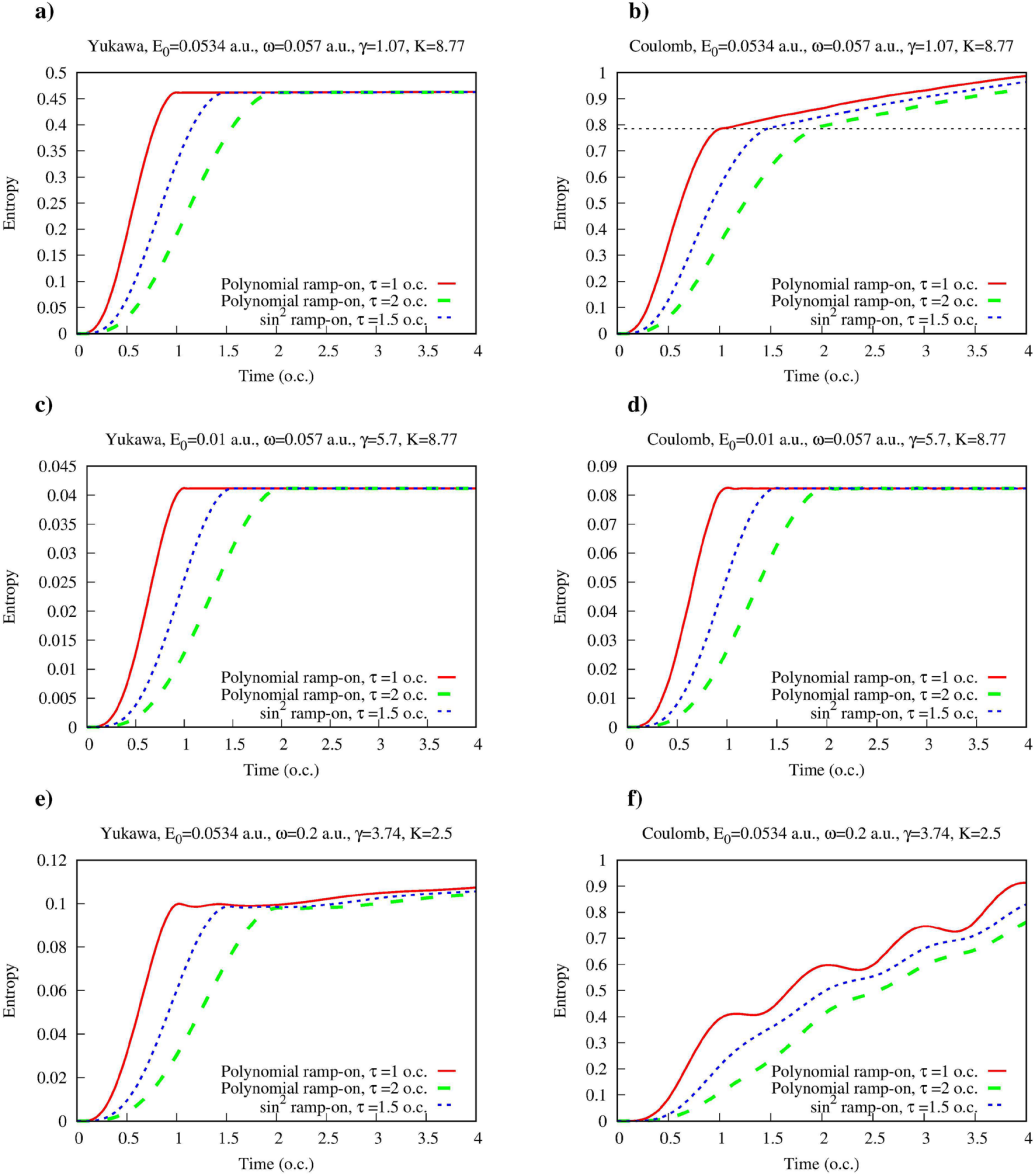
(Color online) Entanglement entropy for the Yukawa and hydrogen atoms in presence of single mode quantized electromagnetic field as a function of time. Time is measured in optical cycles (o.c.) corresponding to the photon frequency $\omega$.

An ingredient of the calculation which we have yet to describe in more detail is the ramp-on function *g*(*t*) in Eq. (2), which describes the switching on of the atom-field interaction. We need a smooth ramp-on function *g*(*t*) to minimize the transient effects. On the other hand, we have to make sure that the particular form of *g*(*t*) that we use, does not affect the physical picture and the conclusions we make. We performed, therefore, calculations with different ramp-on functions *g*(*t*), defined so that $g(0)=0$, $g(t)=1$ for $t> \tau$, where a positive parameter $\tau$ defines duration of the ramping-on of the atom-field interaction. On the interval $(0,\tau )$ we used the polynomial:

10 $$\begin{aligned} g(t)= 3{t^2\over \tau ^2} -2 {t^3\over \tau ^3} \, \end{aligned}$$

and the trigonometric sine-squared:

11 $$\begin{aligned} g(t)= \sin ^2{\left( {\pi t\over 2\tau }\right) } \ \end{aligned}$$

profiles for *g*(*t*). For both profiles given by Eqs. (10) and (11), *g*(*t*) increases monotonously from zero to one on the switching interval $(0,\tau )$. In Fig. 1 we present results we obtain for the Yukawa and hydrogen atoms for various ionization regimes using the different ramp-on functions *g*(*t*). As mentioned above, we characterize the field in the Fock state using the equivalent field strength $E_0$ defined in Eq. (5), and the photon frequency $\omega$. It will also prove convenient below to employ an equivalent set of dimensionless parameters characterizing the field: the Keldysh parameter^21^: $\gamma =\omega \sqrt{2I_p}/E_0$ (here $\omega$, $E_0$ and $I_p$ are the field frequency, field strength and ionization potential of the target atom), and the multiquantumness parameter $K=I_p/\omega$ giving the number of photons needed to ionize the target atom. As it is well-known^21–25,37^, ionization process can proceed in distinctly different ways depending on the value of the Keldysh parameter. For $K \gg 1$ we may have either tunneling $\gamma \lesssim 1$ or multiphoton $\gamma \gg 1$ regimes of ionization. One should note that fulfillment of the condition $K \gg 1$ alone does not necessarily mean that we are dealing with the multiphoton ionization process^38^, the condition $\gamma \gg 1$ should also be satisfied.

In the Fig. 1 we present the results for the multiquantum tunneling regime with $\gamma = 1.07$ and $K=8.77$ (Fig. 1a,b), the multiphoton regime with $\gamma =5.7$ and $K=8.77$ (Fig. 1c,d) regime, and the low-*K* ionization regime with participation of a small number of photons with $\gamma =3.74$, $K=2.5$ (Fig. 1e,f). An observation that one can make upon inspecting the plots in Fig. 1 is that for the Yukawa atom entanglement entropy remains practically constant after the electric field is fully switched on for all the ionization regimes we consider. The entanglement entropy still grows for $t>\tau$ for the Yukawa atom, but in the multiquantum regime (Fig. 1a,c) this growth is so slow that it can hardly be discerned on the plots. For the low-*K* ionization regime of the Yukawa atom shown in (Fig. 1e) this growth is more pronounced, but is still rather slow. Moreover, as one can see, the entanglement entropies obtained for different ramp-on functions *g*(*t*) practically coincide when electric field is fully switched on. This means that for the Yukawa atom, entanglement entropy calculated at the moment $t=\tau$, when the ramping-on of the interaction terminates, provides a well-defined measure of the field-atom entanglement, which does not depend on the particular details of the ramping-on of the atom-field interaction.

For the ionization of the hydrogen atom (Fig. 1b,d,f) dependence of the entanglement entropy on time for $t > \tau$ is more pronounced, especially in the case of the low-*K* ionization regime shown in Fig. 1f. For the more interesting multiquantum regimes shown in Fig. 1b,d this dependence is much weaker than for the low-*K* regime, and entanglement entropy calculated at the moment $t=\tau$ still provides a sensible characteristic of the field-atom entanglement even in the case of the hydrogen atom.

The main features of the behavior of the entanglement entropy can be understood with the help of a more detailed study of the reduced photon density matrix $\hat{\rho }^F$ defined in Eq. (8) which we present in the next Section.

### Properties of the reduced photon density matrix $\hat{\rho }^F$

#### Diagonal and non-diagonal elements of $\hat{\rho }^F$

To get a better understanding of the behavior of the reduced photon density matrix (8), which we need to compute the entanglement entropy in Eq. (9), we will present first a qualitative illustration of the time evolution of the elements of the reduced photon density matrix (8) which we obtain for different field parameters for the Yukawa and hydrogen atoms. In this Section we show the results we obtain for the ramp-on function (10) and $\tau =T$, where $T=2\pi /\omega$- is an optical cycle corresponding to the photon frequency $\omega$. As we saw above, for the moments of time $t>\tau$ the results are not sensitive to a particular choice of the ramping-on function *g*(*t*).

**Figure 2:**
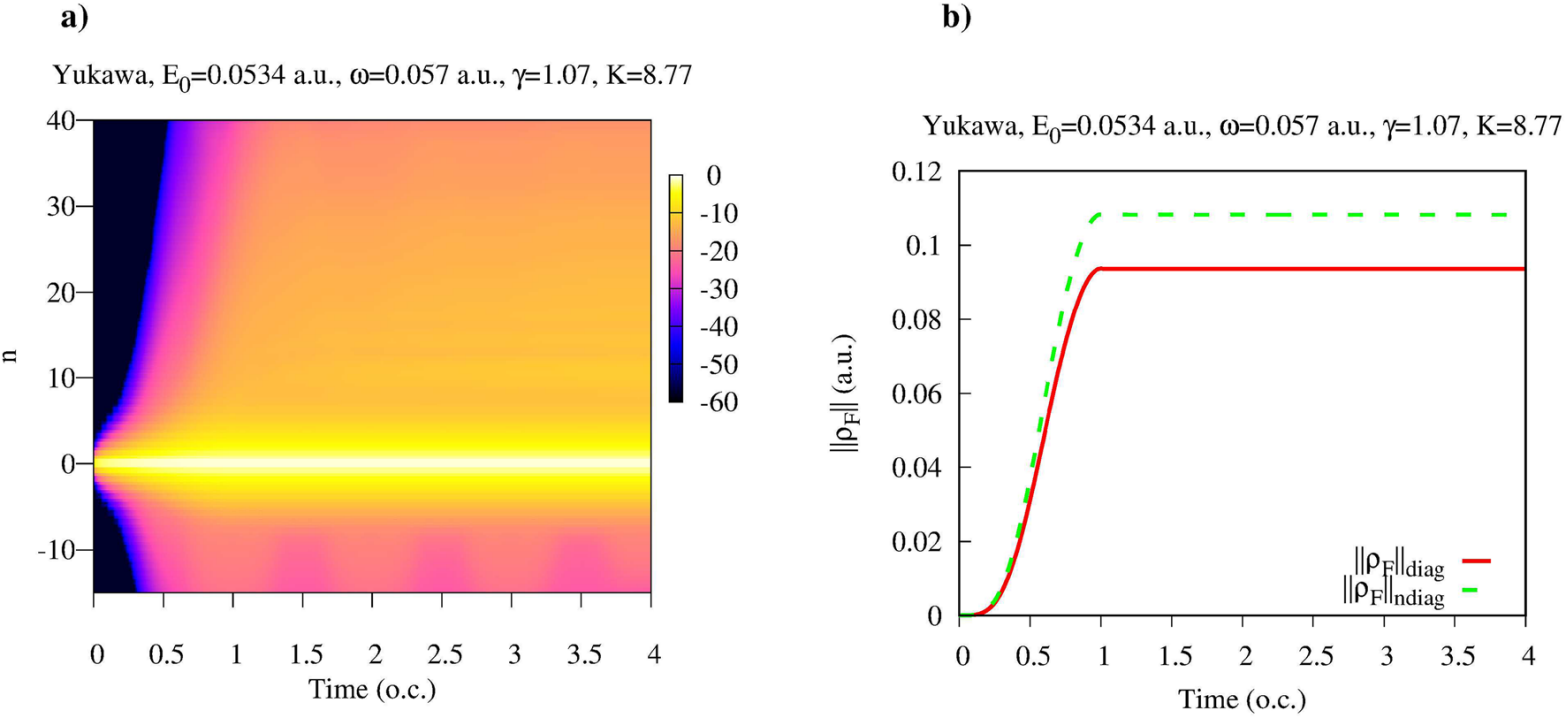
(Color online) (**a**) Diagonal matrix elements $\hat{\rho }^F_{N_0-n,N_0-n}(t)$. Logarithmic scale is used. (**b**) Matrix norms defined in Eq. (12) as functions of time.

In Fig. 2 we show a general picture of the time-evolution of the diagonal elements of $\hat{\rho }^F(t)$ for the Yukawa potential. It is convenient to label the diagonal matrix elements $\rho ^F_{N,N}(t)$ of the matrix (8) as: $\rho ^F_{N_0-n,N_0-n}(t)$, where $N_0$ is the number of photons in the initial state of the electromagnetic field, and *n* an integer. With this definition the diagonal matrix elements $\rho ^F_{N_0-n,N_0-n}(t)$ with $n \ge 0$ represent the probability $P_n(t)$ to find the electron+field system in a state in which the electron absorbed *n* photons from the field at time *t*^1^.

We had to employ logarithmic scale in Fig. 2 since for the field parameters we consider the distribution of the diagonal elements of $\hat{\rho }^F_{N_0-n,N_0-n}(t)$ is by far dominated by $P_0(t)=\hat{\rho }^F_{N_0,N_0}(t)$- the probability that no photons are absorbed or emitted. One can see that $\hat{\rho }^F_{N_0-n,N_0-n}(t)$ have non-negligible values even for negative *n*, although their magnitude is considerably smaller than that of the $\hat{\rho }^F_{N_0-n,N_0-n}(t)$ with positive *n*. With the notation we employ, the matrix element $\hat{\rho }^F_{N_0-n,N_0-n}(t)$ with a negative *n* give us the probability for the atom to emit *n* photons. These matrix elements describe, therefore, the virtual processes in which atom emits *n* photons remaining in the ground state. The presence of such virtual processes does not contradict the energy conservation law since strict energy conservation is obtained in the limit of large evolution times, in agreement with the time-energy uncertainty relation $\Delta E\Delta t \sim 1$^39^.

**Figure 3:**
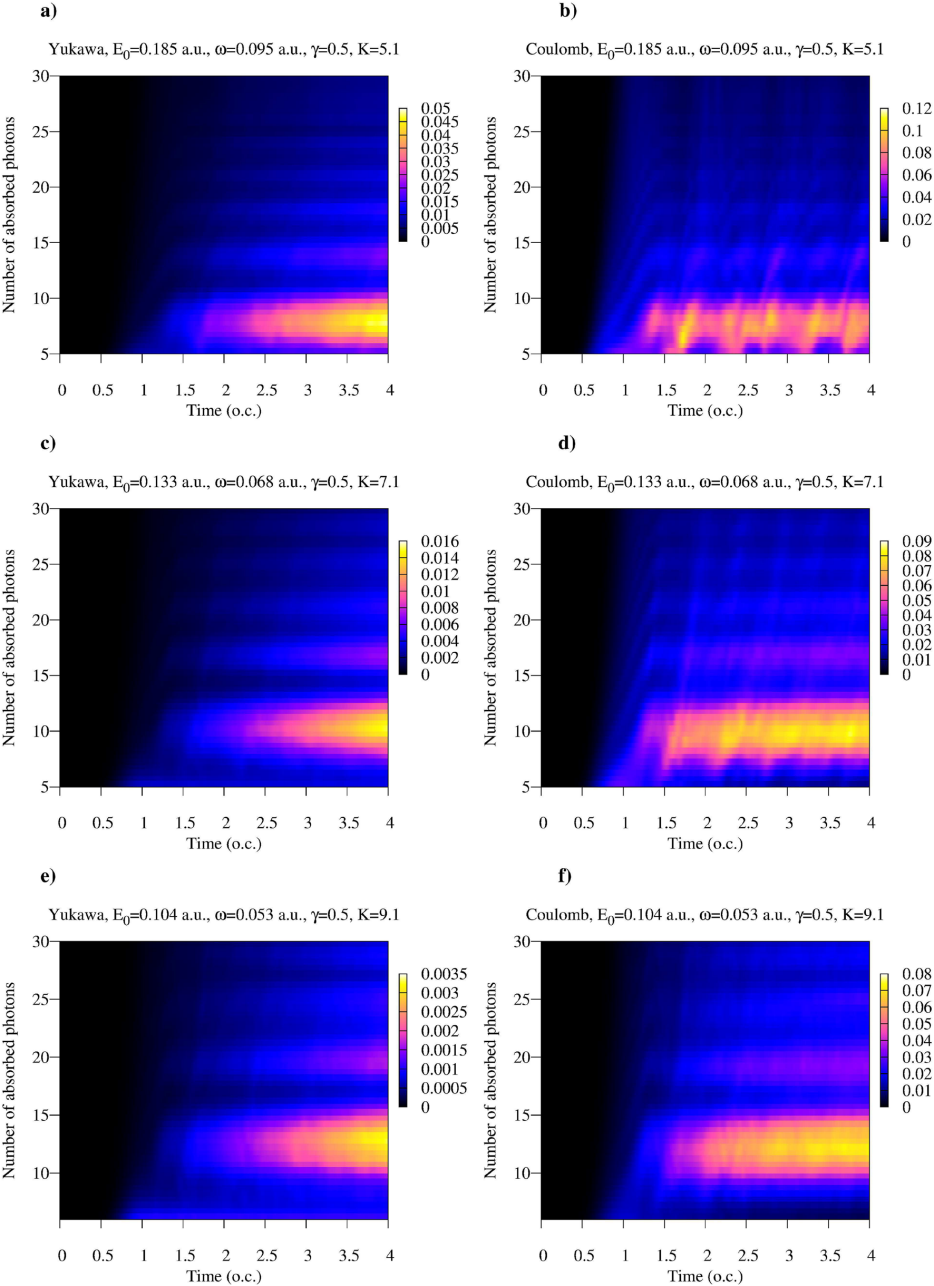
(Color online) Absorption probability as a function of time for the Yukawa and hydrogen atoms for the Keldysh parameter $\gamma =0.5$. Time is measured in optical cycles (o.c.) corresponding to the photon frequency $\omega$.

**Figure 4:**
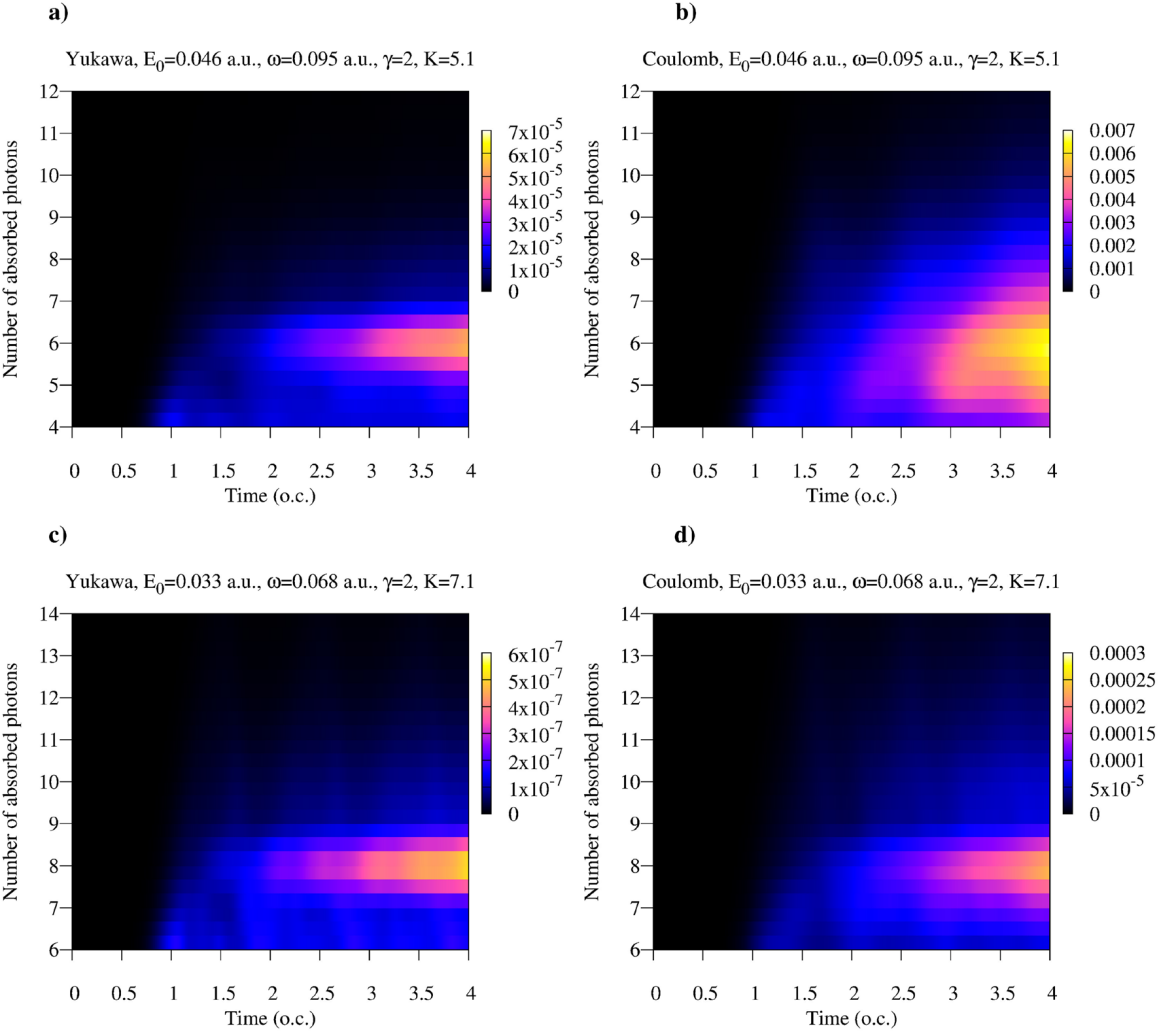
(Color online) Absorption probability as a function of time for the Yukawa and hydrogen atoms for the Keldysh parameter $\gamma =2$. Time is measured in optical cycles (o.c.) corresponding to the photon frequency $\omega$.

A closer look at the distribution of the absorbed photons can be obtained from Figs. 3 and 4, where we show $\hat{\rho }^F_{N_0-n,N_0-n}(t)$ with $n>0$ for various field parameters. We performed a detailed study of the distributions of absorbed photons for the ionization process driven by the quantized electromagnetic field in a Fock state in the work^40^. The results we present in Figs. 3 and 4 agree with the qualitative conclusions we made there. In particular, one may note that the most probable numbers of the absorbed photons in the cases of the Yukawa and Coulomb potentials are approximately equal. On the other hand, the distributions of absorbed photons, which can be obtained by taking vertical slices of the distributions in Figs. 3 and 4 are generally wider for the Coulomb potential. Two mechanisms could account for this effect^40^. In the case of the finite range interaction the motion of the ionized electron is essentially free, and the free electron, as is well known, cannot absorb a photon. In the case of the short range interaction, therefore, the process of the photon absorption is confined to the time interval when electron is inside the range of the short-range potential. Another mechanism which might contribute to a broader distribution of the number of absorbed photons is the photo-excitation process, which is absent in the case of the Yukawa atom which has only one bound state.

The reduced photon density matrix $\hat{\rho }^F$ is by no means defined by its diagonal elements only. To be able to gauge the relative importance of the diagonal and non-diagonal matrix elements of $\hat{\rho }^F$ let us define the following matrix norms:

12 $$\begin{aligned} ||\rho ^F||_{\textrm{diag}}= & {} \left( \sum _{\begin{array}{c} {k} \\ {k\ne N_0} \end{array}} |\rho ^F_{kk}|^2 \right) ^{1\over 2} \nonumber \\ ||\rho ^F||_{\textrm{ndiag}}= & {} \left( \sum _{\begin{array}{c} {k_1 k_2} \\ {k_1\ne k_2} \end{array}} |\rho ^F_{k_1k_2}|^2 \right) ^{1\over 2} \end{aligned}$$

The evolution of the norms introduced in Eq. (12) is shown in Fig. 2b. One can see that $||\rho ^F||_{\textrm{diag}}$ and $||\rho ^F||_{\textrm{diag}}$ are of the same order of magnitude on all the interval of the quantum evolution that we consider.

#### Eigenvalues of the reduced photon density matrix $\hat{\rho }^F$

An analysis of the eigenvalues of $\hat{\rho }^F$ provides more information about the properties of the reduced photon density matrix and the entanglement entropy (9). The reason for this is that, as we shall see, for large values of the multiquantumness parameter *K* only first few eigenvalues of $\hat{\rho }^F$ play important role in Eq. (9) for the entanglement entropy.

The set $\{\lambda _i\}$ of the eigenvalues of the reduced photon density matrix satisfies a few easily deducible conditions. Since, by definition, $\hat{\rho }^F$ is a positive definite Hermitian operator, all its eigenvalues $\lambda _i$ are positive. Moreover, since the reduced photon density matrix satisfies the normalization condition, one must have:

13 $$\begin{aligned} \textrm{Tr}_{\textrm{F}}\hat{\rho }^F(t) = \sum \limits _i \lambda _i=1 \, \end{aligned}$$

where the partial trace operation $\textrm{Tr}_{\textrm{F}}$ consists in tracing over the field variables.

**Figure 5:**
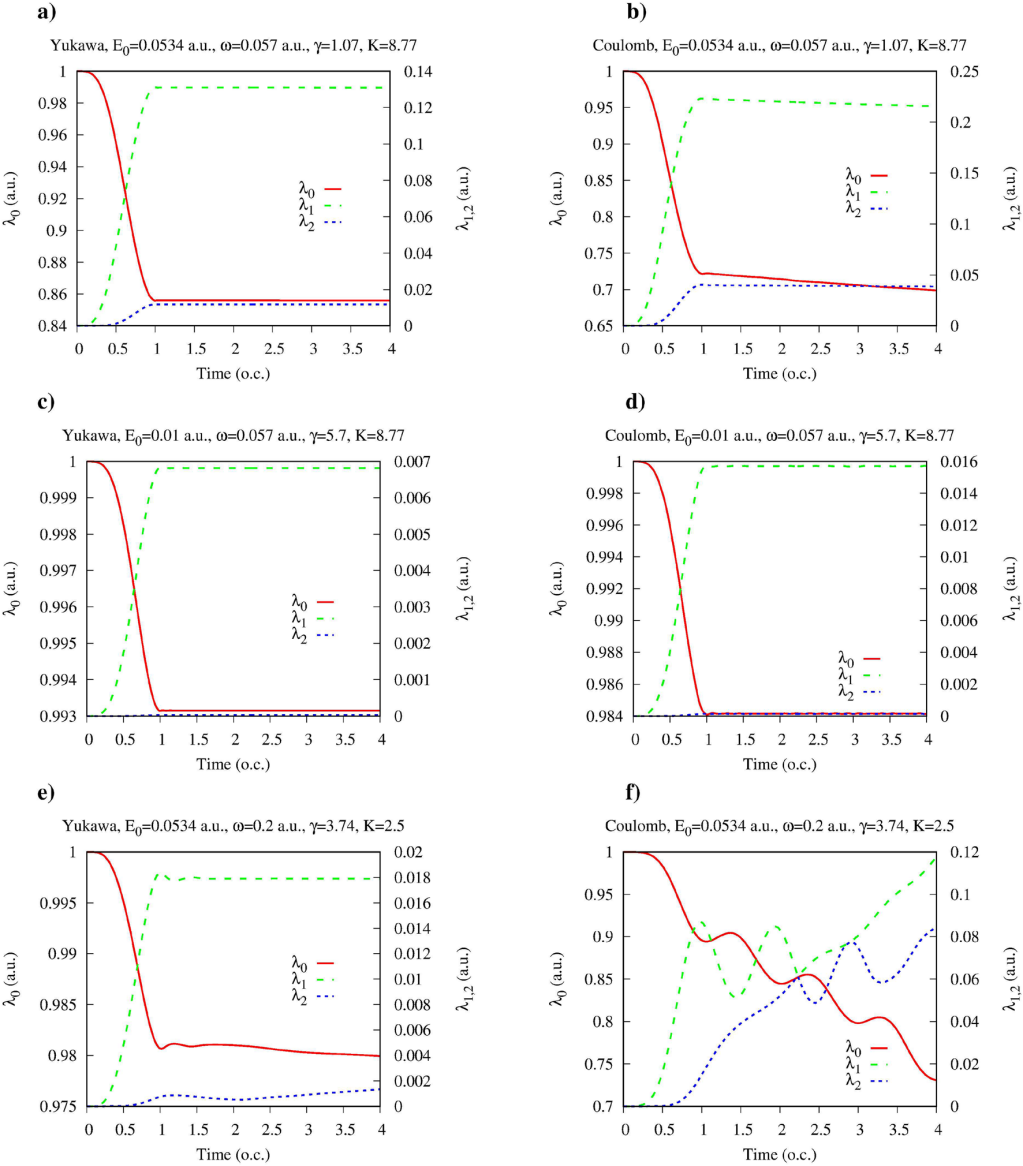
(Color online) The largest eigenvalues of the reduced photon density matrix $\rho ^F(t)$ as functions of time for the Yukawa and hydrogen atoms. Time is measured in optical cycles (o.c.) corresponding to the photon frequency $\omega$.

In Fig. 5 we show the three largest eigenvalues $\lambda _0$, $\lambda _1$ and $\lambda _2$ ($\lambda _2< \lambda _1 < \lambda _0$) as functions of time for the same targets and the same field parameters as in Fig. 1. We see that for the case of the large values of the parameter *K* we have, for both tunneling and multiphoton regimes of ionization, the following ordering of the eigenvalues $\lambda _0 \sim 1 \gg \lambda _1 \gg \lambda _2$. It is clear from the definition (8) of the reduced photon density matrix, that when $|P_0(t)-1|\ll 1$, we must have $\lambda _0(t) \approx P_0(t)$, where $P_0(t)$ is the probability that the atom neither absorbed nor emitted any photons. If we assume that all the eigenvalues $\lambda _i$ with $i>1$ are small and can be neglected in Eq. (9) for the entanglement entropy and in the trace relation (13), than we obtain an estimate $\lambda _1(t)\approx 1-P_0(t)$ from the trace relation, which gives us the following approximate formula for the entanglement entropy:

14 $$\begin{aligned} S= -P_0(t)\log {P_0(t)} - (1-P_0(t))\log {(1-P_0(t))} . \end{aligned}$$

**Figure 6:**
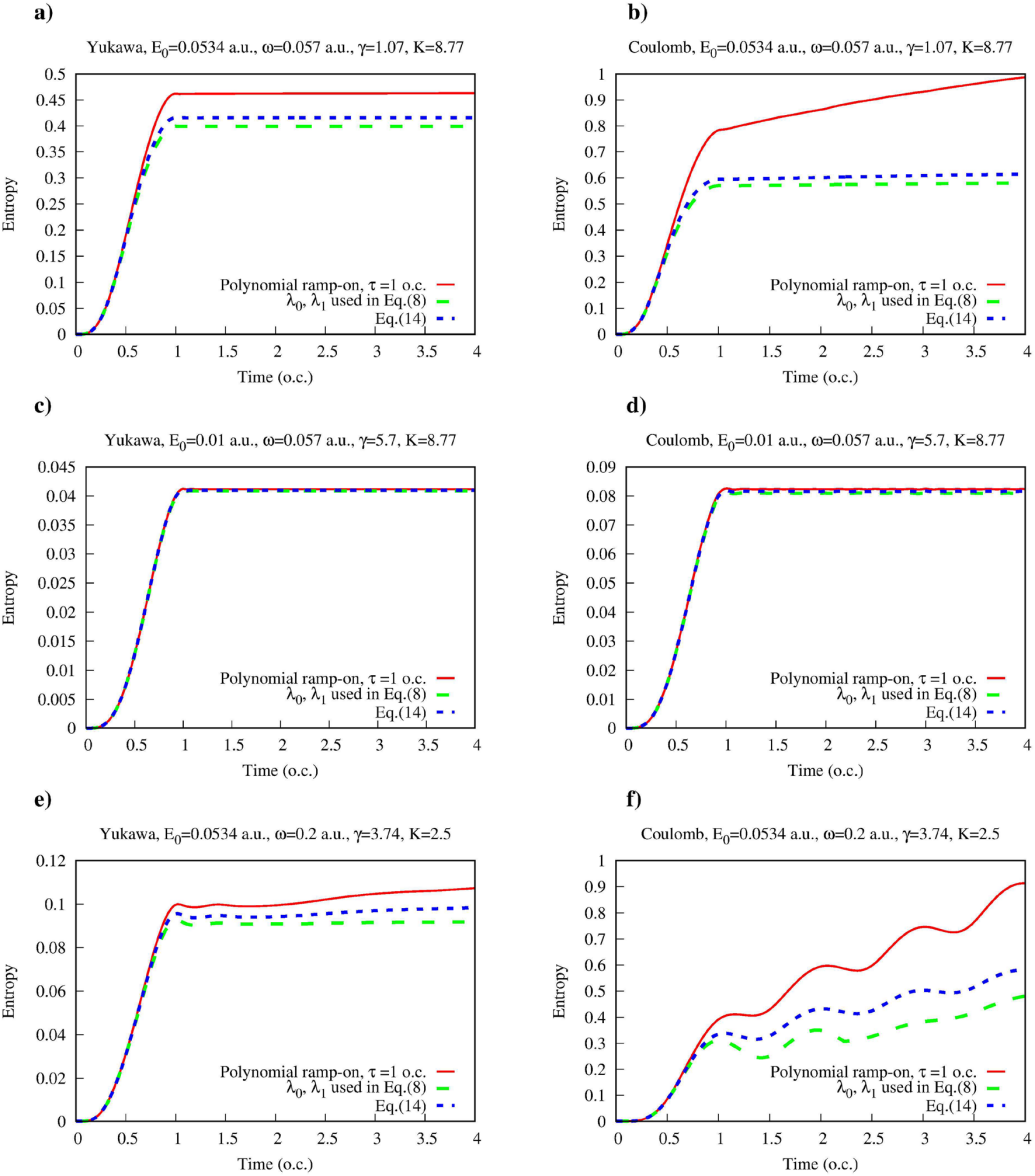
(Color online) Entanglement entropy for the Yukawa and hydrogen atoms in presence of the single mode quantized electromagnetic field as a function of time. (Red) solid line: Eq. (9), (green) dashed line: Eq. (9) with only two greatest eigenvalues $\lambda _0$, $\lambda _1$ kept in the sum, (blue) short dashed line: Eq. (14). Time is measured in optical cycles (o.c.) corresponding to the photon frequency $\omega$.

According to the discussion we presented above, Eq. (14) should be approximately valid for ionization with $K\gg 1$ in both tunneling and multiphoton regimes. The results for the entanglement entropy that we obtain using Eq. (14) are shown in Fig. 6 for the same field parameters and targets shown in Fig. 1. We present results of the exact calculation based on Eq. (9), with all the eigenvalues of $\hat{\rho }^F(t)$ included in the sum, results of an approximation obtained by truncating the sum in Eq. (9) and including only two largest eigenvalues $\lambda _0$, $\lambda _1$ kept in Eq. (9), and the results we obtain using the analytic estimate (14). One can see that by keeping only the terms with the two greatest eigenvalues $\lambda _0$, $\lambda _1$ in Eq. (9), we obtain a good estimate for the entanglement entropy. This fact justifies the assumption we made above that the terms containing the eigenvalues $\lambda _i$ with $i>1$ play relatively minor role in Eq. (9) in the ionization regimes with large *K* values. We see also, that the analytic formula Eq. (14), which follows from this assumption, agrees fairly well with the results of the exact calculation in the large-*K* regimes, and reproduces qualitatively correctly the behavior of the entanglement entropy even in the low-*K* cases shown in Fig. 6e,f.

### Entanglement entropy for different ionization regimes

We demonstrated above, when discussing results shown in Fig. 1, that the entanglement entropies $S(\tau )$ calculated at the moment $t=\tau$ when atom-field interaction is fully switched on, practically do not depend on a particular form of the ramp-on function *g*(*t*). We have seen also that for $t>\tau$ and for the large-*K* ionization regimes, *S*(*t*) is a very slowly growing function of time. It is justified, therefore, to use the entanglement entropy $S(\tau )$ calculated at the moment $t=\tau$ as a quantitative measure of the atom-field entanglement for different ionization regimes provided they belong to the multiquantum large-*K* domain. We will adopt, therefore, $S(\tau )$ as a measure allowing comparison of the entanglement for different ionization regimes. We will present below results of such a comparative study of the entanglement for the ionization regimes with large *K* and arbitrary $\gamma$, in other words, for the tunneling and multiphoton regimes of ionization.

**Figure 7:**
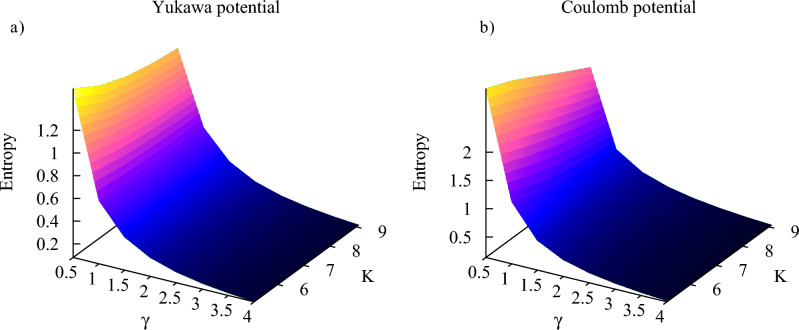
(Color online) Entanglement entropy for different ionization regimes.

**Figure 8:**
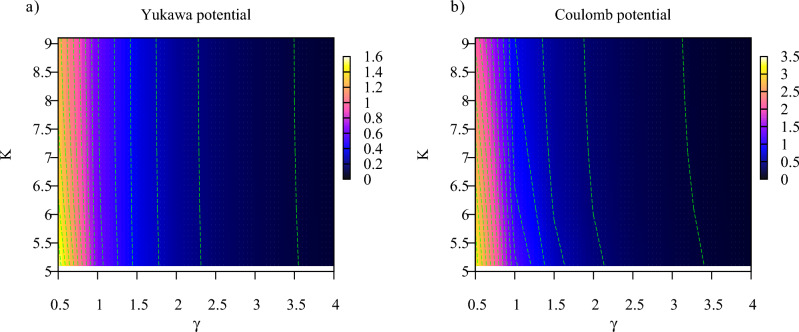
(Color online) Entanglement entropy for different ionization regimes. Contour plot.

In Figs. 7 and 8 we show $S(\tau )$ calculated according to Eq. (9) for different ionization regimes. We used in these calculations the ramp-on function *g*(*t*) given by Eq. (10) with $\tau =T$, where *T* is an optical cycle corresponding to the photon frequency $\omega$. As we discussed above, the results are insensitive to the choice of the particular form of the ramp-on function. For brevity, we will call below $S(\tau )$ the entanglement entropy (understanding, of course, that entanglement entropy still grows, albeit slowly, for $t>\tau$). We confine our study to the multiquantum domain with $K>5$ where, as we saw, $S(\tau )$ provides a reliable characteristic of entanglement, and we present results for the Keldysh parameter $\gamma$ ranging from tunneling ($\gamma =0.5$) to the multiphoton ($\gamma =5$) regimes. Calculations have been performed for both Yukawa and Coulomb systems.

**Figure 9:**
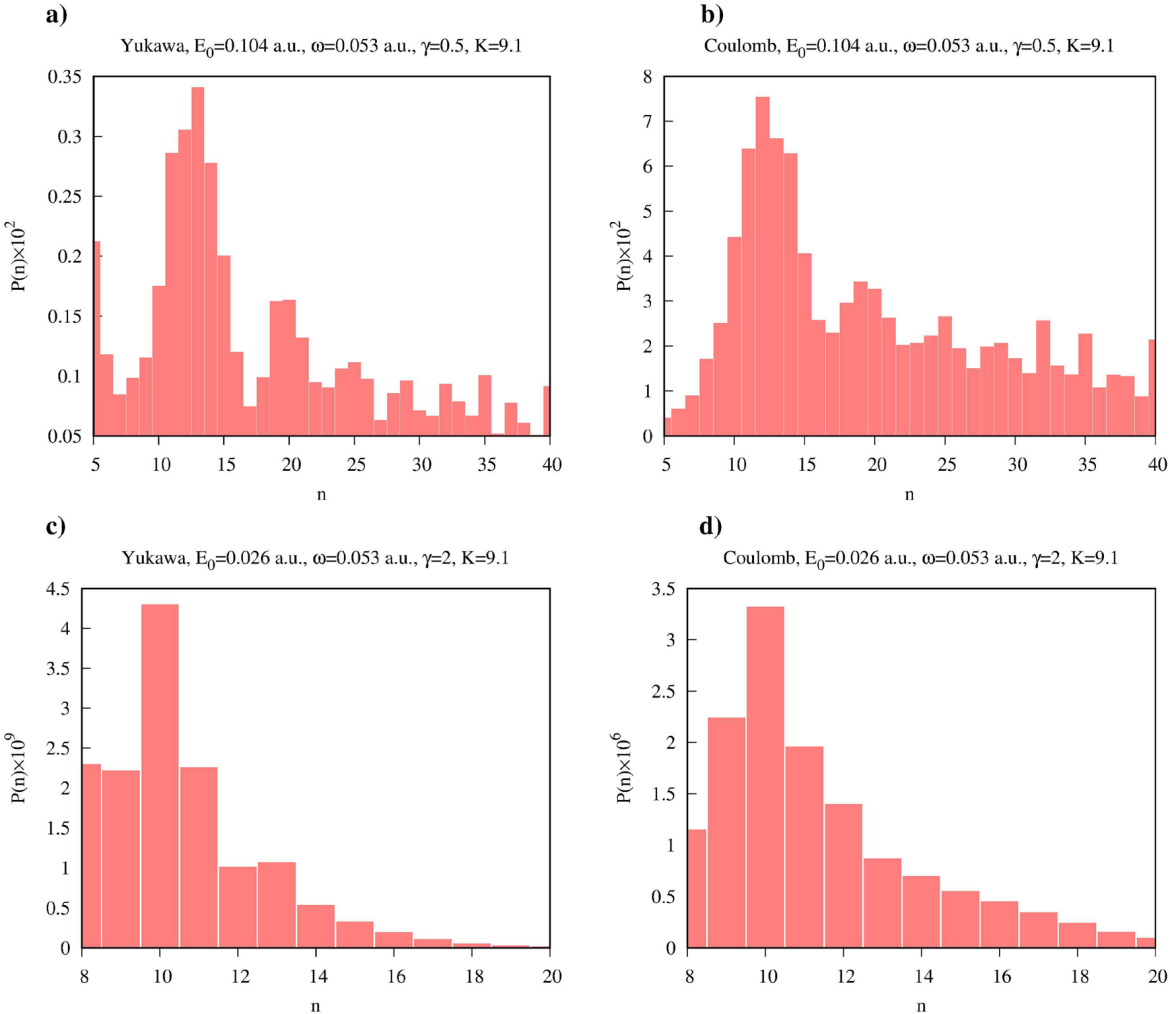
(Color online) Distributions of absorbed photons computed at the moment $t=T$. *T* is an optical cycle corresponding to the photon frequency $\omega$. Ramp-on function (10) with $\tau =T$ has been employed in the calculations.

One can see from the plots that, as a rule, for the same field parameters entanglement entropy is higher in the case of the Coulomb potential. This fact can be explained by recalling the discussion of the reduced photon density matrix we presented above. The distributions of absorbed photons, which can be obtained by taking vertical slices of the distributions shown in Figs. 3 and 4 tend to be wider in the case of the Coulomb interaction. This fact was also noted in the work^40^. As an illustration, we show in Fig. 9 distributions of absorbed photons at $t=\tau$ for the Yukawa and Coulomb cases. Wider photon distributions in the case of the Coulomb potential entail larger entropy.

**Figure 10:**
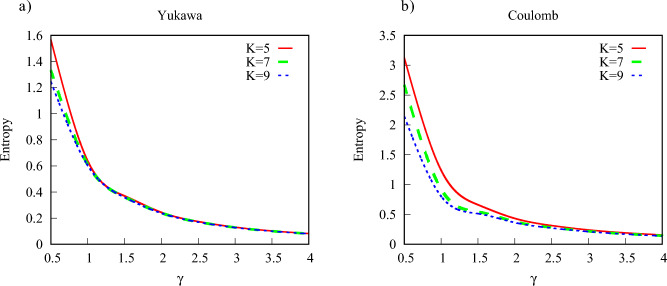
(Color online) Entanglement entropy as a function of $\gamma$ for different *K*.

Another feature which is apparent from Figs. 7 and 8 is the general decrease of the entanglement entropy with increasing *K* for fixed values of $\gamma$, which is most clearly seen in the tunneling regime of small $\gamma$-values. This feature is illustrated in more detail in Fig. 10, where we present entropy as a function of $\gamma$ for several values of *K*. This trend is a consequence of the fact that to keep $\gamma$ constant while decreasing *K*, we have to decrease the photon frequency and proportionally decrease the effective field strength (5). In the tunneling regime this leads to the sharp decrease of the processes corresponding to absorption or emission of photons, so that we have $|1-P_0|\ll 1$ in Eqs. (14), and (14) tells us that the entanglement entropy should decrease. If, on the contrary, we keep *K* (i.e. the photon frequency) constant and increase the value of the $\gamma$ parameter, we gradually move from the tunneling into the multiphoton regime. The decrease of the entanglement entropy in this case can be qualitatively understood by looking at the photon distributions in Fig. 9. Tunneling is a non-resonant process^41^, so we generally have broader photon distributions, and consequently a larger number of terms which contribute significantly in the expansion (6) describing the entangled state, leading to larger values of the entanglement entropy.

### Entanglement entropy on a larger time interval

We have considered so far the situation when atom-field interaction is slowly switched on on the time interval $(0,\tau )$ with the ramp-on function *g*(*t*) in Eq. (2), describing the switching on process, having the following properties: *g*(*t*) is a continuous and never decreasing function of time, $g(0)=0$, $g(t)=1$ for $t> \tau$. An interesting question is what would happen if we switched off the atom-field interaction at some point in the future. On the physical grounds, one would expect that the entanglement entropy should remain constant. These expectations are supported by a simple calculation using the equations of motion for the observables. In the Heisenberg picture we have, for an observable described by the operator ${\hat{O}}$, an equation of motion^39^:

15 $$\begin{aligned} i {d{\hat{O}}\over dt}= \left[ {\hat{H}}, {\hat{O}}(t) \right] \, \end{aligned}$$

For the case ${\hat{O}}= -\hat{\rho }^F(t) \log {\rho ^F(t)}$, where $\rho ^F(t)$ is the reduced photon density matrix, the Hamiltonian operator driving evolution of $\rho ^F(t)$ after atom-field interaction has been switched off, is just the Hamiltonian ${\hat{H}}_F$ of the free electromagnetic field, so:

16 $$\begin{aligned} i {d\over dt} \left( \hat{\rho }^F(t) \log {\rho ^F(t)}\right) = \left[ {\hat{H}}_F, \hat{\rho }^F(t) \log {\rho ^F(t)} \right] . \end{aligned}$$

Taking trace with respect to the photon variables of both sides of this equation, using invariance of trace with respect to the cyclic permutations and recalling definition (9) of the entanglement entropy *S*(*t*), we obtain from Eq. (16): $\displaystyle {dS(t)\over dt}=0$.

As an additional consistency and accuracy check of our calculations, we performed a calculation modeling the situation when atom-field interaction is switched off at some point in the future. We solved the TDSE describing evolution of the bipartite system consisting of the atomic electron and the quantized field on the time interval (0, 8*T*) (where $T=2\pi /\omega$ is an optical cycle corresponding to the field frequency $\omega$) using the following form of the function *g*(*t*) describing the atom-field interaction strength in Eq. (2):

17 $$\begin{aligned} g(t)= \left\{ \begin{array}{l} 3{t^2\over T^2} -2 {t^3\over T^3} \, t\in (0,T) \\ 1 \, t\in (T,6T) \\ 3{(7T-t)^2\over T^2} -2 {(7T-t)^3\over T^3} \, t\in (6T,7T) \\ 0 \, t\in (7T,8T) \end{array} \right. \end{aligned}$$

**Figure 11:**
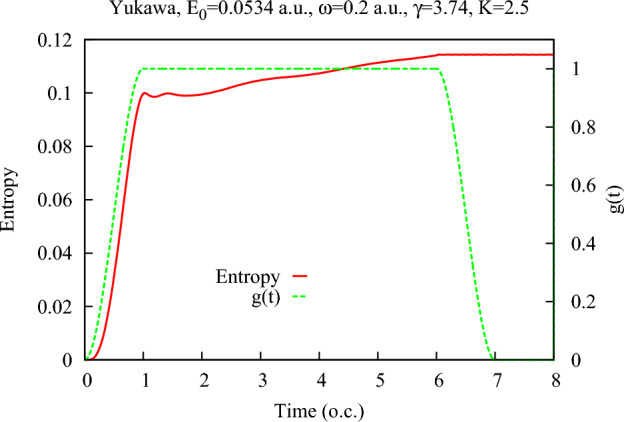
(Color online) Entanglement entropy for the Yukawa atom in presence of the single mode quantized electromagnetic field as a function of time with the function *g*(*t*) in Eq. (2) given by Eq. (17). Time is measured in optical cycles (o.c.) corresponding to the photon frequency $\omega$.

In other words, we use the same switching on procedure as in the case of the ramp-on function (10) with $\tau =T$, and switch off the atom-field interaction symmetrically on the interval $t\in (6T,7T)$. The function *g*(*t*) defined by Eq. (17) is shown in Figs. 11. Figure 11 also shows entanglement entropy as a function of time for the same field parameters and the target atom as in Fig. 1e. One can see that calculated entanglement entropy indeed behaves in agreement with the properties we deduced above using the general arguments. During the process of the electron-field interaction certain amount of entropy is produced, which remains constant after the complete switch-off of the interaction. This behavior of the von Neumann entropy is, to some extent, similar to the case of the free electron interacting with the quantized electromagnetic field considered in^18^. In the case of the initially bound electron that we consider, just as in the case of the free electron studied in^18^, a net entropy production is found on the time interval when the electron and the field interact. The photon and the electron subsystems, therefore, always end up in an entangled state after the moment when the electron-photon interaction is switched off.

## Conclusions

We performed a study of the entanglement between electron and quantized electromagnetic field subsystems for the process of ionization of the Yukawa and hydrogen atoms. Our study was based on the numerical *ab initio* solution of the TDSE describing quantum evolution of the bipartite system consisting of the atom and the quantized electromagnetic field.

Using solution of the TDSE we calculated the reduced photon density matrix $\hat{\rho }^F$, which was subsequently used to compute the entanglement entropy. We were able to explain some properties of the entanglement entropy by analyzing $\hat{\rho }^F$ and its eigenvalues. The approximate Eq. (14) obtained as a result of this analysis, was shown to reproduce satisfactorily behavior of the entanglement entropy in both multiphoton and tunneling regimes of ionization.

We have shown that in the ionization regimes, characterized by large values of the mutiquantumness parameter *K*, the entanglement entropy is a slowly varying function of time on the time interval where atom-field interaction is switched on completely. Moreover, the value of the entanglement entropy at the moment of time $\tau$, when the switching on of the atom-field interaction terminates, does not depend on the particular form of the ramp-on function describing details of the switching process. This observation allows to use the entanglement entropy calculated at the moment $t=\tau$ as a quantitative characteristics of the atom-field entanglement and allows a comparative study of the entanglement for different ionization regimes with large values of the multiquantumness parameter *K*.

Such a comparative study showed that for the same set of the field parameters, entanglement entropy for the hydrogen atom is larger than for the Yukawa atom, which can be attributed to the effects of the long range Coulomb force on the ionized electron. We have shown also that for a fixed value of the Keldysh parameter $\gamma$ and increasing multiquantumness parameter *K*, and for a fixed *K* and increasing $\gamma$, entanglement entropy decreases. We gave qualitative explanation of this behavior of the entanglement entropy on the basis of the analysis of the properties of the reduced photon density matrix.

We have considered the case of the quantized electromagnetic field prepared in the Fock state. Fock states are highly non-classical states for which the photon number operator ${\hat{N}}_{{\varvec{k}},\lambda }={\hat{a}}^{\dag }_{{\varvec{k}},\lambda }{\hat{a}}_{{\varvec{k}},\lambda }$ in the mode ${\varvec{k}},\lambda$ has a definite value. Consequently, in these states the conjugate variable, i.e. the phase of the field^1^, is completely undefined. A question arises, therefore, to what extent the present results are applicable for the case of the atomic ionization driven by the commonly used laser pulses. Quantum state of the electromagnetic field for such a pulse can be well modeled by a coherent state of the field^30^:

18 $$\begin{aligned} |v\rangle = \exp ^{-{|v|^2\over 2}}\sum \limits _{N=0}^{\infty } c_N|N\rangle \, \end{aligned}$$

where $c_N={v^N\over \sqrt{N}}$, $v=|v|e^{-i\phi }$ is an arbitrary complex number. Unlike the Fock states we have considered above, the coherent states have non-zero expectation values of the field operators. In particular, form Eqs. (2) to (18) one obtains for the vector potential for the one-mode case we consider presently: $\displaystyle \langle v|\hat{{\varvec{A}}}({\varvec{r}},t)|v\rangle = \sqrt{8\pi |v|^2 c^2\over \omega V} {\varvec{e}}_{z} \cos {(\omega t-{\varvec{k}}\cdot {\varvec{r}}+\phi )}$.

We examined the case of the coherent initial state of the field and compared atomic ionization driven by the field in the Fock state and in the coherent state in the work^40^. This study was based on a semiclassical representation of the quantized electromagnetic field proposed in^42^. This approach can be used when the number of photons in the Fock state is $N_0\gg 1$. This theoretical procedure is described in detail in the Section Methods below. The results obtained in^40^ show that the atomic characteristics, such as the spectra of the ionized electrons, ionization probabilities, etc., are virtually identical for the calculations using coherent initial states and the Fock initial state with the same equivalent field strength defined as prescribed by the Eq. (5). This fact was implicitly understood for a long time. The first studies of the statistical distributions for the number of absorbed photons in the process of the strong field ionization were published long time ago^25,43^. These studies were based on the standard SFA approach, which uses completely classical description of the electromagnetic field^25,43^. The paper^43^, in particular, relying on the standard SFA approach with the field considered entirely classically, discusses the non-Poissonic character of the statistical distributions of absorbed photons for the process of strong field ionization. We presented in the work^40^ a detailed comparison of the results for the distributions of the number of absorbed photons obtained using the SFA based approach^25,43^, and the results obtained using the approach relying on the semiclassical quantum treatment of the electromagnetic field developed in^42^. The results proved to be qualitatively quite similar, with some minor differences which can be attributed to the approximate character of the SFA method. The semiclassical theory developed in^42^ explains the reason for this as follows.

In the framework of the theoretical approach proposed in^42^ the effect of the totally undefined phase of the field in the Fock state can be accounted for by performing a suitable averaging of the electron density matrices obtained for the classical monochromatic waves with the same field strength and different carrier envelope phases (CEP) (the averaging prescription is given by the Eq. (30) below). For the long pulses the CEP effects fade and can be neglected, Therefore, the reduced electron density matrices obtained for the cases of the Fock and coherent initial states give virtually identical results for the statistical distributions of various observables. Entanglement entropy was calculated in the present work using Eq. (8) and expressing it in terms of the reduced photon density matrix. As Eq. (7) shows, we could use for this purpose the reduced electron density matrix, obtaining the same result. We saw also that the entanglement entropy becomes a very slowly growing function of time after the moment when the atom-field interaction is switched on completely. We can expect, therefore, that on the long time intervals, when the CEP effects can be neglected, our results for the entanglement entropy obtained for the initial Fock state of the field, should be applicable for the case of the initial coherent state of the field.

All the calculations above were performed for the case when only a single mode $({\varvec{k}},\lambda )$ of the electromagnetic field is present. Another aspect in which the present procedure might be generalized or modified is the case when several modes of the electromagnetic field have to be included in the consideration. This might be necessary, for instance, for the study of the pulse shape effects, where the effect of the pulse envelope can be represented by including additional modes in the expansion (2). Yet more interesting could be a study of the high harmonic generation (HHG) for the atom driven by the field in a Fock state, which would necessitate inclusion of the additional modes corresponding to the emission of harmonic photons. From the purely theoretical point of view, the present numerical procedure that we used to solve the TDSE for the bipartite system could be modified relatively easily for the multi-mode case. The only obstacles we could meet moving in this direction are of purely computational character. Computational cost of our numerical procedure is roughly the same as the cost of the numerical procedures used to solve the TDSE for atomic systems described in the Single Active Electron Approximation, driven by the classical elliptically polarized electric field. With heavy parallelization, the time for such a calculation to complete on a supecomputer is usually in the order of several hours. Adding more modes would linearly increase the computing time making such a calculation computationally costly, but still feasible. We did not need to include additional modes for the present calculation. We neglected, in particular, all the modes we would need to describe emission of the harmonic photons. In this respect, our procedure is similar to the semiclassical procedure proposed in^42^ which is briefly described below in the Methods Section. An essential feature of this procedure is a convenient representation of the annihilation and creation operators obtained in the limit when $N_0\rightarrow \infty$, $|m| \ll N_0$ in Eq. (20). From the physical point of view taking this limit corresponds to the neglect of the processes of spontaneous photon emission or absorption and account of only the stimulated photon emission or absorption processes, which essentially coincides with the physical content of our approach. Mathematically, the neglect of the spontaneous emission processes corresponds to the neglect of the terms of the order of $1/N_0$ in the dynamic equations^42^. For the typical field frequency $\omega$ and the equivalent field strength $E_0$ that we consider, $N_0$-values are in the range of $10^5$–$10^6$ (the procedure we use to determine $N_0$ is discussed in detail in the subsection Numerical Solution of the TDSE of the Section Methods), which certainly makes permissible the neglect of the terms of the order of $1/N_0$.

An interesting question is that of the possibility of experimental observation and measure of the atom-field entanglement. For an entangled state of a bipartite system, the reduced density matrices of both subsystems represent mixed rather than pure states^1^. The reduced density matrix of any of the subsystems can be used for the calculation of the entanglement entropy. To detect entanglement one can, therefore, analyze the reduced density matrix characterizing one of the subsystems. For the case in question of the bipartite system consisting of atom and quantized photon field it is easier to analyze the reduced density matrix describing the electron subsystem after the moment of time when the atom-field interaction has been switched off. Such an analysis, allowing to reconstruct the density matrix by performing a set of incompatible measurements on a system, can be done using the methods of quantum tomography^44^. It is important to note that such a measurement is meaningful only when atom-field interaction has been completely switched off. During the interval of the pulse duration the separation of the bipartite system in the electron and the field subsystems is, strictly speaking, unphysical. Indeed, such a separation depends on the gauge used to describe interaction of the electron and the quantized field. Instead of using the minimal coupling interaction Hamiltonian in Eq. (4) we might use, for instance, the Goppert-Mayer gauge, which is a generalization of the well-known quantum-mechanical length gauge for the case of the quantized electromagnetic field. Corresponding Hamiltonian could be obtained from the Hamiltonian (4) by means of the Goppert-Mayer transformation^45^, which would affect reduced density matrices describing photon and electron subsystems. This transformation would, therefore, generally affect the entanglement entropy (9) for the times inside the laser pulse duration. Only when atom-field interaction is switched off and entanglement entropy ceases to vary, one obtains results independent of the gauge.

## Methods

### Numerical solution to the TDSE

We use the coordinate representation for the vectors $|f_N(t)\rangle$ in Eq. (6). We omit, therefore Dirac notation for these vectors and will write them simply as functions $f_N({\varvec{r}},t)$ of coordinates and time. Just as in the case of the ordinary TDSE, which treats the atom-field interaction classically^31–33^, we represent $f_N({\varvec{r}},t)$ as a series in spherical harmonics (we assume that the field given by Eq. (2) is polarized in *z*-direction):

19 $$\begin{aligned} f_N({\varvec{r}},t) = \sum \limits _{l=0}^{l_{\textrm{max}}} f_{Nl}(r,t) Y_{l0}({\varvec{n}}) . \end{aligned}$$

The radial variable *r* is treated by discretizing the TDSE on a grid with a step-size $\delta r=0.05$ a.u. in a box of the size $R_{\textrm{max}}$. The initial ground state of the system was obtained by using a variational calculation employing the Slater basis set^46,47^ with subsequent propagation in imaginary time^48,49^ on the spatial grid we described above.

By substituting expansions (6) and (19) into the TDSE (4), projecting the result on the vectors $Y_{l_i0}({\varvec{n}}) \otimes |N_i\rangle$ with different $l_i$, $N_i$, and using the formulas (22) for the matrix elements, one obtains system of coupled differential equations for the functions $f_{Nl}(r,t)$. This system of differential equations has been solved using a matrix iteration method^50^. Computationally, this procedure is quite similar to the procedure employed for the solution of the ordinary TDSE^31–33^.

Parameters $n_1$, $n_2$ in Eq. (6) and parameter $l_{\textrm{max}}$ in Eq. (19) were chosen to ensure convergence of these expansions. A rule of thumb we used when choosing the values for the parameters $n_1$ and $n_2$ was that they should exceed the maximum number of photons which can be absorbed (parameter $n_1$) or emitted (parameter $n_2$) during the evolution. To choose parameters $l_{\textrm{max}}$ and $R_{\textrm{max}}$ properly, we used the values which were known to produce accurate results from the previous experience of solving ordinary TDSE for the classical field with strength related to the photon number $N_0$ according to the relation (5). Thus, for the equivalent field strength $E_0=0.0534$ a.u. and the photon frequency $\omega =0.057$ a.u., the choice of $R_{\textrm{max}}=500$ a.u., $l_{\textrm{max}}=50$, $n_1=n_2=50$ allows to obtain convergent results for the atom-field evolution on the interval (0, 4*T*), where $T=2\pi /\omega$ is an optical cycle corresponding to the frequency $\omega$.

In practical calculations we have to fix the value of the volume *V* in Eq. (2) for the quantized vector potential. We choose this volume to be a box with the size of $10^3$ a.u. For a given equivalent field strength we then determine the value of $N_0$ using Eq. (5). For the range of the frequencies and the equivalent field strengths that we consider, $N_0$ thus defined is in the range of $10^5$–$10^6$. For instance, for $E_0=0.0534$ a.u. (corresponding to the intensity of $10^{14}$ W/cm^2^) and $\omega =0.057$ a.u. (wavelength of 800 nm) we obtain $N_0=1990525$.

### Semiclassical approach

We describe in this Section a semiclassical method^42^ for the description of quantum evolution of the bipartite system consisting of the atom and the quantized electromagnetic field. The method is particularly illuminating since it demonstrates a simple connection between the quantum and classical treatments of the electromagnetic field. The method is based on the following realization of the Fock space. This representation proposed in^42^ consists in mapping the Fock states $|N_0+m\rangle$ in Eq. (6) on the set of exponential functions of angle $\theta$:

20 $$\begin{aligned} |N\rangle = |N_0+m\rangle = e^{im\theta } \, \end{aligned}$$

Under this mapping the photon part of the Hilbert space ${{\mathscr {H}}}_{\textrm{field}}$ becomes a Hilbert space of functions $f(\theta )$, defined on the interval $\theta \in (0,2\pi )$, with the scalar product:

21 $$\begin{aligned} \langle f|g\rangle ={1\over 2\pi } \int \limits _0^{2\pi } f^*(\theta )g(\theta )\ d\theta . \end{aligned}$$

The creation and annihilation operators become operators acting on the functions depending on $\theta$ as follows:

22 $$\begin{aligned} a= & {} e^{-i\theta }\left( N-i{\partial \over \partial \theta }\right) ^{1\over 2} \nonumber \\ a^{\dag }= & {} \left( N-i{\partial \over \partial \theta }\right) ^{1\over 2} e^{i\theta } \end{aligned}$$

It is easy to see that this representation agrees with the usual properties $a|N\rangle =\sqrt{N}|N-1\rangle$, $a^{\dag }|N\rangle =\sqrt{N+1}|N+1\rangle$ of the creation and annihilation operators.

Representation (22) is an exact mapping. If we assume that in Eq. (20) $N_0\rightarrow \infty$, $|m| \ll N_0$, i.e., we are interested in strong enough fields and the processes with not too large numbers of absorbed of emitted photons, Eq. (22) simplifies to:

23 $$\begin{aligned} a= & {} e^{-i\theta } \sqrt{N_0} \nonumber \\ a^{\dag }= & {} e^{i\theta }\sqrt{N_0} \end{aligned}$$

Using these asymptotic relations the quantized vector potential (2) can be written in the limit $N_0\rightarrow \infty$ as:

24 $$\begin{aligned} \hat{{\varvec{A}}}({\varvec{r}},t)= \sqrt{2N_0\pi c^2\over \omega V} \left( {\varvec{e}}_z e^{-i\omega t-i\theta } + h.c. \right) . \end{aligned}$$

One should note that taking the asymptotic large $-N_0$ limit in Eq. (23) one discards certain processes, such as spontaneous emission or absorption of photons. These processes acquire an additional factor of $1/N_0$ comparing to the stimulated emission or absorption processes, and cannot, therefore be described using the leading order of the $1/N_0$ expansion. The processes of the stimulated emission and absorption which are of interest to us in the present work, on the other hand, can be considered in the leading order of $1/N_0$-expansion. The expression in (24) has a form of the classical expression for the vector potential. One should realize, however^42^, that it is an operator in the photon sector of the Hilbert space ${{\mathscr {H}}}_{\textrm{field}}$ because of the dependence on the angle $\theta$.

The quantum electrodynamic (QED) time-evolution propagator ${\hat{U}}(t,0)$ driving evolution of the bipartite system consisting of atomic electron and quantized electromagnetic field in the combined Hilbert space ${{\mathscr {H}}}_{\textrm{el}}\otimes {{\mathscr {H}}}_{\textrm{field}}$ can be written in a closed form using the Dyson time-ordering operator $\hat{\mathscr {T}}$^51^:

25 $$\begin{aligned} {\hat{U}}(t,0) = \hat{\mathscr {T}} \exp {\left( -i\int \limits _0^t{\hat{H}}(t')\ dt'\right) } \, \end{aligned}$$

where $$\displaystyle {\hat{H}}(t)= {\hat{H}}_{\textrm{el}}+ {{\hat{{\varvec{A}}}}\hat{{\varvec{p}}}\over c} + {{\hat{{\varvec{A}}}}^2\over 2c^2}$$

Using the representation (20), expression (24), and the definition (21) of the scalar product in ${{\mathscr {H}}}_{\textrm{field}}$, one can see that in the limit of large $N_0$ the matrix elements of the QED time-evolution propagator ${\hat{U}}(t,0)$ calculated in the basis of the Fock states can be written as^42^:

26 $$\begin{aligned} \left\langle N_0+m \left| {\hat{U}}(t,0) \right| N_0 \right\rangle = {1\over {2\pi }}\int \limits _0^{2\pi } {\hat{U}}(t,0;\theta ) e^{-im\theta } \ d\theta . \end{aligned}$$

here ${\hat{U}}(t,0;\phi )$ is an operator acting in the electron Hilbert space ${{\mathscr {H}}}_{\textrm{el}}$ only. This operator satisfies the familiar time-dependent Schrödinger equation^42^:

27 $$\begin{aligned} i{\partial {\hat{U}}(t,0;\theta )\over \partial t}= \left( {\hat{H}}_{\textrm{el}} + {1\over 2c}\hat{{\varvec{p}}}{{\varvec{A}}(\theta )}+ {1\over 2c}{{\varvec{A}}(\theta )}\hat{{\varvec{p}}} + {1\over 2c^2}{{\varvec{A}}(\theta )}^2 \right) {\hat{U}}(t,0;\theta )\, \end{aligned}$$

where ${\varvec{A}}(\theta )$ is now a classical field:

28 $$\begin{aligned} {{\varvec{A}}}({\varvec{r}},t,\theta )= \sqrt{2N\pi c^2\over \omega V} \left( {\varvec{e}}_z e^{-i\omega t-i\theta } + h.c. \right) = {\varvec{A}}_0\cos {(\omega t + \theta )} \, \end{aligned}$$

with the amplitude $\displaystyle A_0= \sqrt{8N\pi c^2\over \omega V}$. All we have to do, therefore, to find the matrix elements of the complete QED propagator in the basis of the Fock states is to find the electron propagator ${\hat{U}}(t,0;\theta )$ in Eq. (27) as a function of the phase $\theta$, and to calculate the integral in the Eq. (26). Using this prescription and acting with the QED propagator (26) on the initial state of the bipartite system, one obtains the following expression for the vectors $|f_N(t)\rangle$ from the electron Hilbert space ${{\mathscr {H}}}_{\textrm{el}}$ in the expression (6) for the wave-function of the system:

29 $$\begin{aligned} |f_{N_0+m}(t)\rangle = {1\over {2\pi }}\int \limits _0^{2\pi } {\hat{U}}(t,0;\theta ) e^{-im\theta }\phi _0 \ d\theta . \end{aligned}$$

Another formula that one can derive from the Eqs. (20) and (29) is the representation for the electron density matrix at time *t*, which can be obtained by taking partial trace with respect to photon degrees of freedom, with the result^42^:

30 $$\begin{aligned} \hat{\rho }_{\textrm{el}}(t)= & {} \sum \limits _m \langle N+m|\Phi (t)\rangle \langle \Phi (t)|N+m\rangle \nonumber \\= & {} {1\over 2\pi } \int \limits _0^{2\pi } {\hat{U}}(t,0;\theta )\hat{\rho }_{\textrm{el}}(0) {\hat{U}}^{\dag }(t,0;\theta )\ d\theta \, \end{aligned}$$

where $\hat{\rho }_{\textrm{el}}(0)=|\phi _0\rangle \langle \phi _0|$ is the initial electron density matrix, and ${\hat{U}}(t,0;\theta )$ is the electron propagator (27) describing evolution driven by the monochromatic field (28).

Equations (29) and (30) demonstrate that the time evolution of the bipartite system consisting of the atom and quantized electromagnetic field prepared in the Fock state, can be modeled by solving ordinary quantum-mechanical TDSE describing evolution driven by the classical monochromatic field (28) with different phases $\theta$. All one has to do is to solve the TDSE and perform certain “phase-average” as prescribed by Eqs. (29), (30). From the physical point of view this recipe is quite natural. This “phase-average” takes into account the well-known fact, that since Fock state of the field is characterized by the fixed definite number of photons, the conjugate variable, i.e. the phase of the field^1^, is completely undefined.

## Data Availability

All data generated or analyzed during this study are included in this published article.
